# EXPOSE-R2: The Astrobiological ESA Mission on Board of the International Space Station

**DOI:** 10.3389/fmicb.2017.01533

**Published:** 2017-08-15

**Authors:** Elke Rabbow, Petra Rettberg, Andre Parpart, Corinna Panitz, Wolfgang Schulte, Ferdinand Molter, Esther Jaramillo, René Demets, Peter Weiß, Rainer Willnecker

**Affiliations:** ^1^Institute of Aerospace Medicine, Radiation Biology, German Aerospace Center Cologne, Germany; ^2^Institute of Pharmacology and Toxicology, Uniklinik RWTH Aachen Aachen, Germany; ^3^OHB System AG Wessling, Germany; ^4^RUAG Schweiz AG, RUAG Space Zürich, Switzerland; ^5^European Space Research and Technology Centre, European Space Agency Noordwijk, Netherlands; ^6^Microgravity User Support Center, German Aerospace Center Cologne, Germany

**Keywords:** astrobiology, ground simulation, survival, UV, vacuum, extra-terrestrial UV radiation, temperature, space missions

## Abstract

On July 23, 2014, the Progress cargo spacecraft 56P was launched from Baikonur to the International Space Station (ISS), carrying EXPOSE-R2, the third ESA (European Space Agency) EXPOSE facility, the second EXPOSE on the outside platform of the Russian Zvezda module, with four international astrobiological experiments into space. More than 600 biological samples of archaea, bacteria (as biofilms and in planktonic form), lichens, fungi, plant seeds, triops eggs, mosses and 150 samples of organic compounds were exposed to the harsh space environment and to parameters similar to those on the Mars surface. Radiation dosimeters distributed over the whole facility complemented the scientific payload. Three extravehicular activities later the chemical samples were returned to Earth on March 2, 2016, with Soyuz 44S, having spent 588 days in space. The biological samples arrived back later, on June 18, 2016, with 45S, after a total duration in space of 531 days. The exposure of the samples to Low Earth Orbit vacuum lasted for 531 days and was divided in two parts: protected against solar irradiation during the first 62 days, followed by exposure to solar radiation during the subsequent 469 days. In parallel to the space mission, a Mission Ground Reference (MGR) experiment with a flight identical Hardware and a complete flight identical set of samples was performed at the premises of DLR (German Aerospace Center) in Cologne by MUSC (Microgravity User Support Center), according to the mission data either downloaded from the ISS (temperature data, facility status, inner pressure status) or provided by RedShift Design and Engineering BVBA, Belgium (calculated ultra violet radiation fluence data). In this paper, the EXPOSE-R2 facility, the experimental samples, mission parameters, environmental parameters, and the overall mission and MGR sequences are described, building the background for the research papers of the individual experiments, their analysis and results.

## Introduction

Space environment provides a variety of environmental extreme parameters that up to date cannot be simulated on ground. In particular the combination of low pressure, temperature oscillations, short wavelength UV (ultraviolet) and the complex cosmic ionizing radiation is only available in space and provides a research environment for astrobiology that resembles other solar system bodies and moons closer than laboratories on Earth can. One of the first opportunities for astrobiological samples to be exposed to space conditions was provided by Apollo 16 on its round trip to moon in 1972 (Bucker et al., 1973, 1974). Since Apollo, a variety of international astrobiological exposure experiments utilizing the space UV regime with short wavelength <295 nm cut off by the ozone layer on Earth, the ionizing radiation climate consisting of combinations of protons, electrons, and HZE [High Z (= atomic number) Energy] particles and microgravity were performed on retrievable and non-retrievable satellites as well as on space stations, developed by Space Agencies in United States, Europe, and Russia and reviewed in several papers (Taylor et al., 1974; Horneck, 1998a; Horneck et al., 2010; Raggio et al., 2011). Retrievable experiment platforms in Low Earth Orbit (LEO) allowing the postflight analysis of the experiments on ground were accommodated, e.g., on (i) space shuttles, as the German Exposure tray mounted 1983 on the cold plate of the cargo bay of Spacelab SL1 (Horneck et al., 1984a) and 1993 of Spacelab D2 (Horneck et al., 1996), (ii) retrievable free-flying satellites like the NASA Long Duration Exposure Facility (LDEF, 1984–1990) with the German Exostack (Horneck et al., 1994), the ESA (European Space Agency) Exobiology Radiation Assembly (ERA) flown 1992–1993 on the EUropean REtrievable CArrier (EURECA) (Horneck et al., 1995; Baglioni et al., 2007) and the satellites Bion-9 (Cosmos-2044, 1989), Bion-10 (1992/93), Bion-11 (1996) (Kuzicheva and Gontareva, 2003), Bion-M1 (2013) and five ESA BIOPAN missions (1994–2007) flown with Russian Foton satellites (Demets et al., 2005), (iii) space stations like the exposure platforms on Russian space missions that included the space stations Salyut-7 and MIR, the French “Exobiologie” experiment flown 1999 on the Russian MIR station (Rettberg et al., 2002) and the BIORISK canisters on the International Space Station (ISS) from IBMP/Roscosmos (2005–2009). Recently, non-retrievable free flying carriers of the CubeSat series equipped with *in situ* measurement systems like the NASA O/OREOS mission^1^ (Woellert et al., 2011) indicate a new era of future active astrobiological experiments.

Access to space and its environment provides the opportunity to investigate the effects of extreme environmental conditions on life, supporting research on resistance strategies and underlying metabolic mechanisms, on the astrobiological questions of the origin and evolution of life, the limits of life and the habitability of other solar system bodies and the possible distribution of life beyond Earth. Many biological samples were exposed in astrobiological space missions, from microorganisms to small animals, lichen, and plant seeds (Horneck et al., 1984b, 2010; Horneck and Brack, 1992; Horneck, 1998b; Sancho et al., 2007; Jonsson et al., 2008). Focus was laid on the exploration of the lithopanspermia scenario, a hypothetic natural way of interplanetary transfer of life (Nicholson et al., 2000; Horneck et al., 2001; de La Torre et al., 2010; Raggio et al., 2011). Access to space also increases our knowledge on the interstellar, cometary, and planetary chemistry and its role in the origin of life (Kuzicheva and Gontareva, 2001, 2002; Cottin et al., 2008) and on the chemical modification, evolution, survival, and destruction of complex organics like polycyclic aromatic hydrocarbons, fullerenes and complex aromatic networks in outer space (Ehrenfreund and Foing, 1997). Recently, beginning in 2008, three consecutive astrobiological exposure missions were conducted on the ISS, carrying biological and chemical samples alike to space in the retrievable ESA multiuser exposure facilities EXPOSE (Rabbow et al., 2012, 2015).

## The EXPOSE-R2 Facility Hardware

The design of EXPOSE is based on the heritage of the Space Shuttle missions SL1 and D2 exposure platforms and ERA of the EURECA mission (Rabbow et al., 2009). ESA initiated the ERA Follow-on Scientific Study under ESA Contract no. 8116/88/F/BZ (SC) (Horneck et al., 1988) and as consequence developed the ESA-EXPOSE multiuser exposure facilities for long-term exposure of international and interdisciplinary experiments to the space environment on the outside of the ISS. The facility was designed following the scientific requirements to provide access to space environment, in particular (i) different UV regimes with respect to wavelength spectrum (i.e., Space, Mars) and transmitted irradiance (attenuation), (ii) space vacuum or other planetary atmospheres and pressures, (iii) temperature oscillation, with (iv) constant real time monitoring, and (v) telemetry of the environmental conditions and the facility health and status. In addition EXPOSE was required to facilitate long duration exposure in space for as many samples of different sizes as possible and the return of the samples to ground for analysis.

The EXPOSE core flight hardware (HW) was a box-shaped structure of 480 mm × 390 mm area and 140 mm height, and a mass of approximately 44 kg. It was equipped with four UV sensors (CUV 280 sensor, OEC GmbH, Germany), one on each corner of EXPOSE. In addition, one radiometer (Dexter 6M Thin Film Based Thermopile Detector) monitored the full solar electromagnetic spectrum^2^. A temperature sensor at the Reference Point of the core HW initiated the survival heaters at temperatures below -25°C to keep EXPOSE electronics operating. Three trays were inserted into the upper part of the box-shaped structure. Each tray measured 466 mm × 120 mm × 72 mm (length × width × height). The final mass of each tray was 5–5.5 kg. Four rectangular cavities of 77 mm × 83 mm and 33.5 mm depth, called compartments, accommodated stacks of two or three sample carriers. Each tray was equipped with a venting line connecting the compartments with the tray’s valve (originally designed by Phitec, Switzerland). Valves opening and closing was commanded from ground. On the bottom side of trays 1 and 2, three temperature sensors were attached (type S 651 PDX 24 B by MINCO Europe, France). On the bottom of tray 3, two Minco sensors as in trays 1 and 2 were attached, and one AD590 sensor by Analog Devices. Plugs connected the trays with auxiliary electronics in the bottom part of the box-like structure. Together with the adapters for attachment to the external ISS platform on the Russian Zvezda Module it was called “monoblock.”

The here described EXPOSE-R2 mission was the third EXPOSE mission, reusing the monoblock from EXPOSE-R, the first mission on the URM-D platform outside of the Russian Zvezda module, flown from March 2009 to February 2011. After 682 days exposure to the LEO environment, the complete EXPOSE-R facility was returned into the ISS, but only the three trays loaded with the scientific samples were returned to Ground at the end of the 833 days mission. The monoblock with the UV and radiation sensors, electronics and heaters remained stored inside the ISS. Therefore, the UV sensors could not be re-calibrated. An overview of the EXPOSE-R mission (Rabbow et al., 2015) and results of the experiments are presented in the same special issue of the International Journal of Astrobiology (Volume 14, Issue 1, 2015).

**FIGURE 1:**
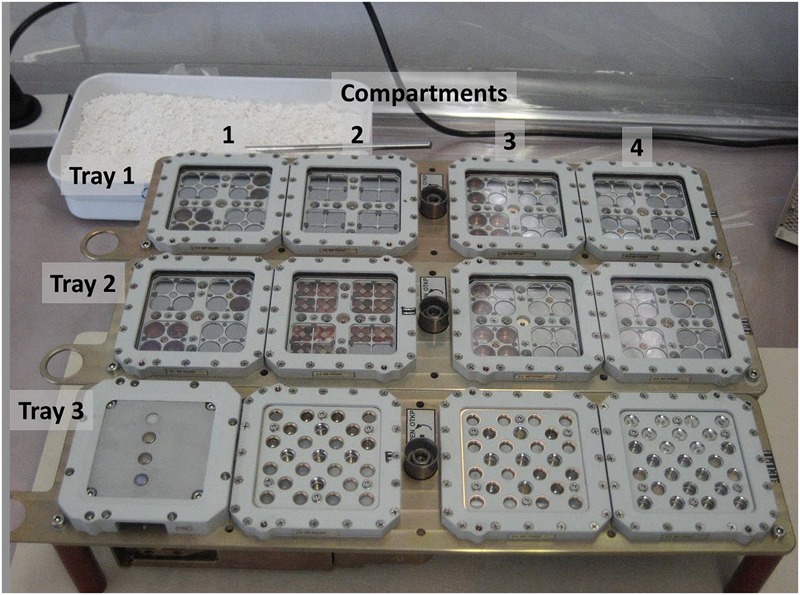
EXPOSE-R2 flight trays with all experiments integrated. Top to bottom: Trays 1, 2, 3; left to right: compartments 1, 2, 3, and 4. Valves and earrings are on the left side. Bottom left compartment with the active radiation measurement instrument R3D. The earrings were intended to secure the trays during EVAs. Credit: DLR.

The compartments of each tray were numbered starting at the valve side of the tray and—for EXPOSE-R and -R2—the earring intended to secure the trays during extravehicular activities (EVAs) (**Figure 1**). An overview of the EXPOSE-E mission in 2008 and results of the experiments are presented in a special collection of Astrobiology 2012. EXPOSE-R2 environmental and status data were collected every 10 s and stored as well as down linked as telemetry. Data of the mission were made available to the investigators and the public on the MUSC (Microgravity User Support Center) web page^3^ and used for the mission parallel Mission Ground Reference (MGR) experiments in the Planetary and Space Simulation Facilities (PSI) at DLR (German Aerospace Center) in Cologne, Germany^4^.

**FIGURE 2:**
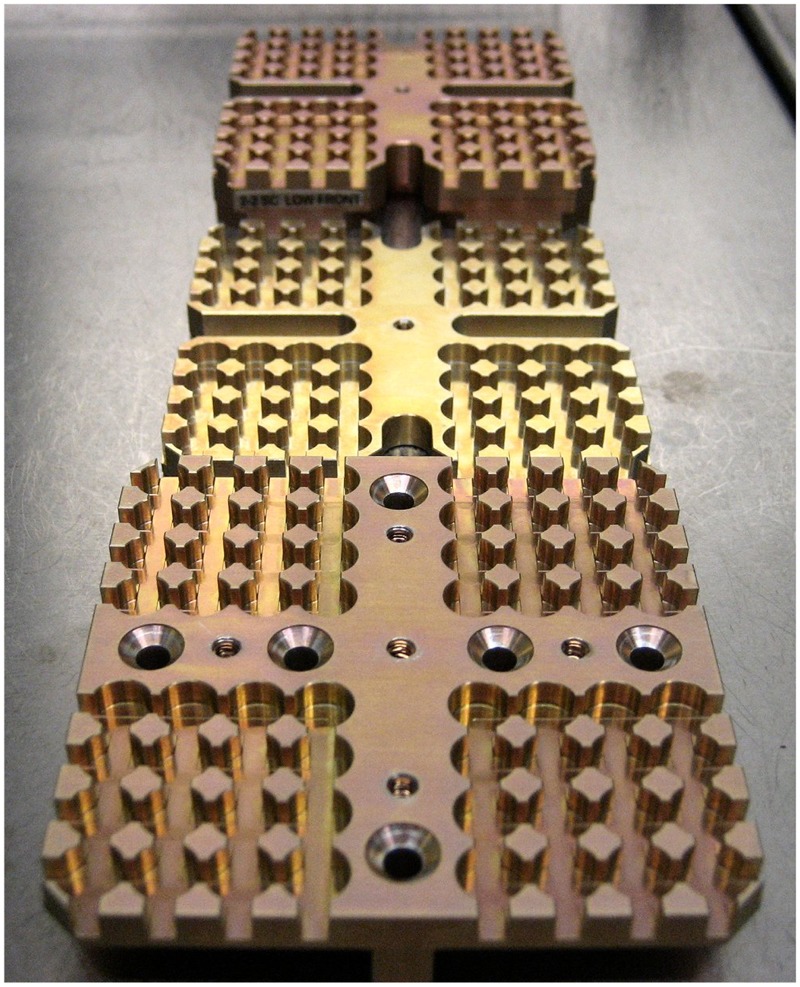
Sixty-four-well sample carrier 2-2, three layers, from top to bottom: low, intermediate, and up carrier. Credit: DLR.

**FIGURE 3:**
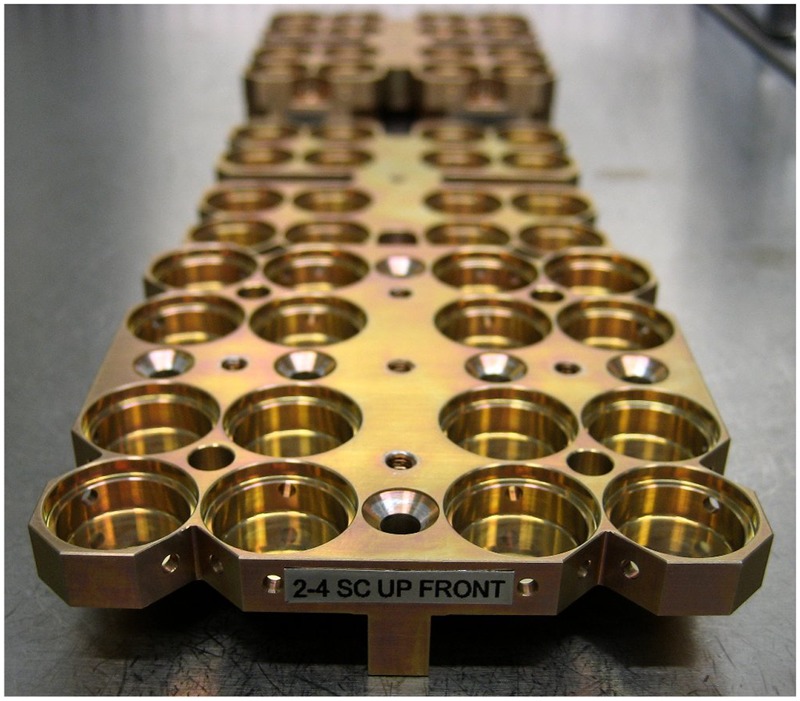
Design of 16-well sample carriers 2-4 with three layers: top, middle, and bottom, marked up, intermediate, and low. Credit: DLR.

**FIGURE 4:**
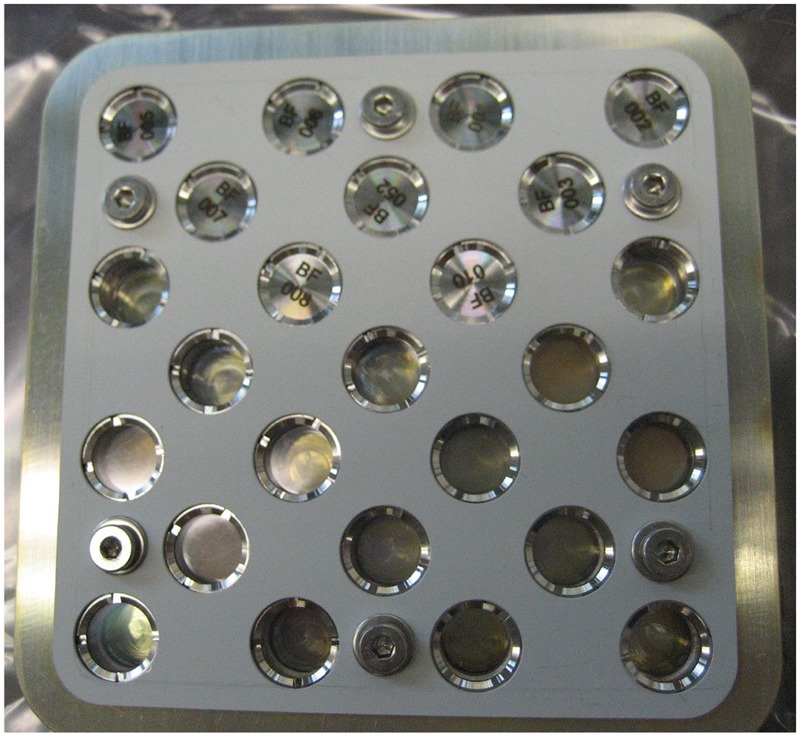
Design of 25 cell top carrier of tray 3 P.S.S. experiment. Credit: Cottin, LISA.

The biological samples were accommodated in stacks of two or three sample carriers into the compartments of the trays 1 and 2. Depending on the type, the sample carriers provided space for 64 samples of 7 mm diameter and 10 mm height (**Figure 2**) or for 16 samples of maximum 12 mm diameter and 4 mm height (for three layers, **Figure 3**) or 6 mm height (for two layers) in sample wells. The chemical samples in 25 individual sample containers called “cells” were integrated in two stacked sample carrier layers each in three compartments of tray 3 (**Figure 4**). This stacked conformation of the sample carriers allowed a completely identical exposure of all samples in one compartment to the space conditions except solar UV radiation. Only the samples in the top carriers were irradiated while the sample in the lower carriers were completely shaded, providing the respective in-flight dark controls. Each sample hole of the 16-well biological sample carriers was covered with an individual 2 mm thick magnesium fluoride (MgF_2_) (tray 1) or quartz (tray 2) sample window. On top of each carrier, filter frames with two layers of selected cut off and neutral density (ND) filters were accommodated to attenuate the solar UV radiation. MgF_2_ glassware allowed transmission of extraterrestrial short wavelength UV radiation with wavelength >120 nm and was accommodated in tray 1 (MolTech GmbH, Germany). For the simulation of a Mars-like UV spectrum with wavelength >200 nm in tray 2, quartz windows and filters were complemented by long pass cut off filters of approximately 50% transmission at 216 nm (MolTech GmbH, Germany; coated by ThinFilmPhysics, Switzerland). Individual wavelength independent UV attenuation for the EXPOSE-R2 samples was achieved by additional 0.1 or 0.001% transmission ND filters made of MgF_2_ or quartz, respectively (MolTech GmbH, Germany; coated by ThinFilmPhysics, Switzerland). Each compartment was covered by an 8 mm thick optical MgF_2_ (tray 1) or quartz (tray 2) top window (MolTech GmbH, Germany).

Tray 3 sample cells screwed into the sample carriers of the chemical experiment were individually closed by 1 mm thick MgF_2_ windows to minimize UV transmission loss. The compartments accommodating the astrochemical experiment P.S.S. were closed by the upper sample carriers themselves. Top windows and the tray 3 upper carriers were sealed gas tight to the trays with window frames and O-rings screwed to the structure with 25 screws each. Only the first compartment of tray 3 was not connected to the venting line. This compartment was powered for the insertion of the active dosimetry instrument R3D-R2, a radiation sensor package for UV and ionizing radiation measurements. While the dosimeter was secured in the compartments also by a respective window frame, it was not sealed.

## EXPOSE-R2 Mission Ground Reference Hardware

In parallel to the space mission, an MGR experiment was performed at DLR, Cologne, Germany, using its PSI. A mission similar set of trays was provided to accommodate and expose a full flight parallel set of samples to simulated space parameters as similar to those experienced by the flight samples in space as was technically feasible. The MGR trays were identical to the flight trays except that they were not powered. Therefore, they were not equipped with temperature sensors, the valves were operated manually and no active dosimeter R3D-R2 was foreseen, leaving the respective compartment 3-1 empty.

## The Astrobiological Experiments on EXPOSE-R2

**FIGURE 5:**
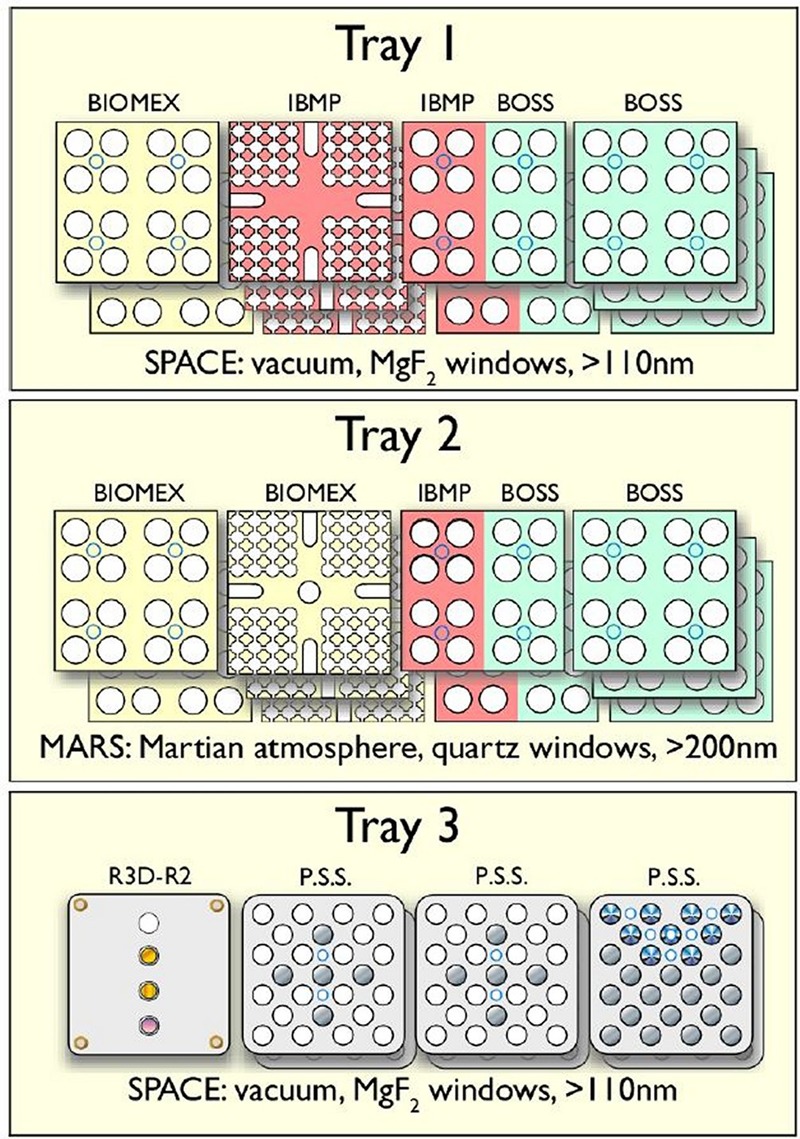
Experiments as accommodated for flight in EXPOSE-R2. Credit: René Demets, ESA.

**FIGURE 6:**
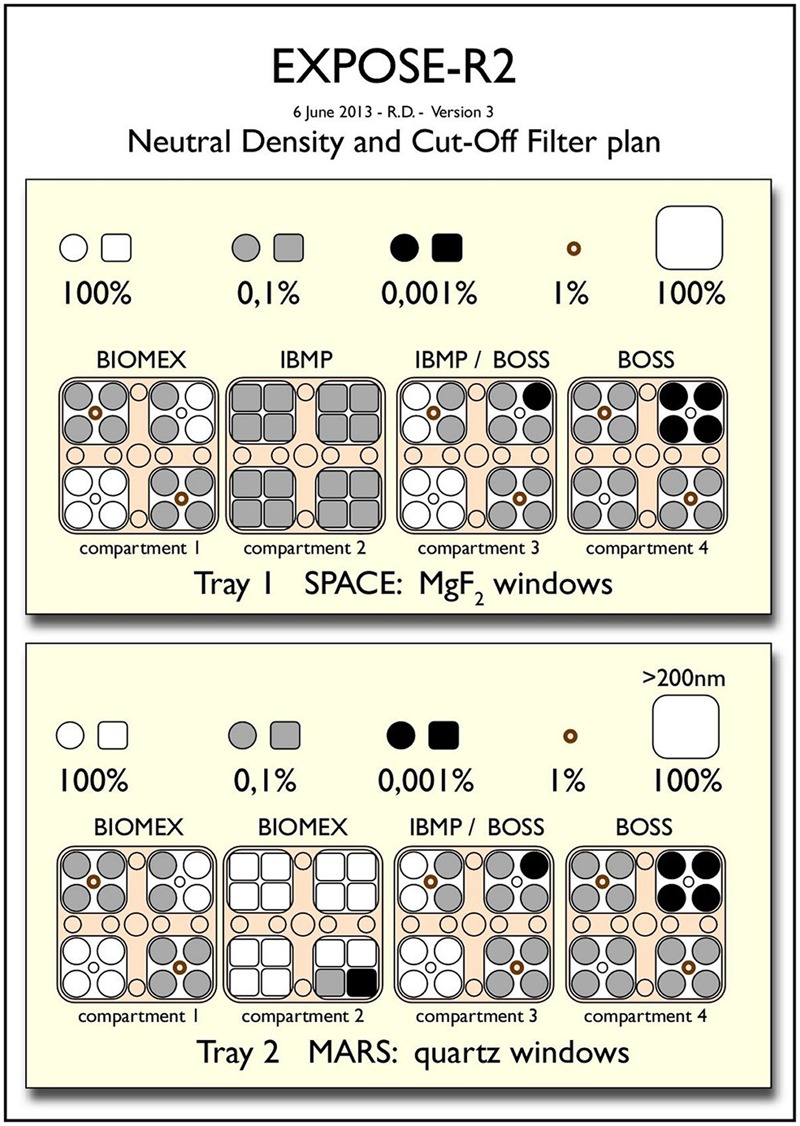
Filter frame with integrated optical filters for trays 1 and 2. In the top left and bottom right quarters, PPO stacks covered by small round neutral density filters are integrated (brown circles). Credit: René Demets, ESA.

For the EXPOSE-R2 mission, three international experiments proposed in response to the ESA ILSRA 2009 Announcement of Opportunity were selected: two biological experiments BIOMEX [principal investigator (PI) J.-P. de Vera, DLR, Berlin, Germany] and BOSS (PI P. Rettberg, DLR, Cologne, Germany) and the organic chemical experiment P.S.S. (PI H. Cottin, LISA, Paris, France). In addition, the Russian biological experiment BIODIVERSITY was provided by IBMP, Moscow (Russia). Results of the experiments are expected to be published in Frontiers in Microbiology under the Research Topic Title: Habitability Beyond Earth and in an upcoming special collection of Astrobiology. The three ESA experiments were accommodated in three compartments each, while two compartments were allocated to the IBMP experiment. The remaining active compartment was reserved for the R3D-R2 instrument. The experiments, investigators, affiliations, countries, and the respective flown samples are collected in **Table 1**. The final numbers of samples per experiment are summarized in **Table 2**. The distribution of the experiments in EXPOSE-R2 is shown in **Figure 5** and an overview of the optical filters is given in **Figure 6**. All compartments except the active and completely separate R3D-R2 compartment in tray 3 were connected to the venting line of the respective trays, therefore open to the atmospheric conditions outside of the tray via the valve.

**Table 1:** List of EXPOSE-R2 experiments, investigators, and samples.

Experiment	Investigator	Affiliation	Samples
BIOMEX 1	D. Billi	University of Roma 2, Italy	*Chroococcidiopsis* CCMEE 029
BIOMEX 2	N. Kozyrovska	Kyiv, Ukraine	*Paenibacillus* and *Kombucha*
BIOMEX 3	S. Onofri	University Tuscia, Viterbo, Italy	*Cryomyces antarcticus*
BIOMEX 4	Th. Leya	Fraunhofer IBMT, Potsdam, Germany	*Chlamydocapsa* CCCryo 101-99 and *Nostoc commune* CCCryo 231-06
BIOMEX 5	R. de la Torre	INTA, Madrid, Spain	*Circinaria gyrosa*
BIOMEX 6	N. Feyh	TU Berlin, Germany	Biofilm
BIOMEX 7	P. Rettberg	DLR, Cologne, Germany	*Deinococcus radiodurans*
BIOMEX 8	S. Ott	University of Düsseldorf, Germany	Pigments and *Buellia frigida*
BIOMEX 9	B. Huwe	University of Potsdam, Germany	*Grimmia sessitana*
BIOMEX 10	D. Wagner	AWI Potsdam, Germany	*Methanosarcina* sp.
BIOMEX 11	C. Cockell	University of Edinburgh, Scotland, United Kingdom	*Gloeocapsa* OU20

BOSS 1	P. Rettberg (PI)	DLR, Cologne, Germany	*Deinococcus geothermalis*, biofilms and planktonic
BOSS 2	C. Cockell	University of Edinburgh, Scotland, United Kingdom	*Gloeocapsa* OU 20 biofilm
BOSS 3	S. Leuko H. Stan-Lotter	DLR, Cologne, Germany University of Salzburg, Austria	*Halomonas* salina, *Halococcus morrhuae salinarum* biofilms, planktonic and mixture of both
BOSS 4	K. Venkateswaran	JPL, Pasadena, United States	*Bacillus horneckiae*, spores
BOSS 5	D. Billi	University of Roma 2, Italy	*Chroococcidiopsis*, 029 and 057 biofilms and planctonic

BIODIVERSITY 1	V. Sychev (PI)	IBMP, Moscow, Russia	Variety of bacteria, fungi, plant seeds
BIODIVERSITY 2	O. Gusev	National Institute of Agrobiological Sciences, Tsukuba, Japan	*Anhydrobiotic chironomid* larvae
BIODIVERSITY 3	M. Sugimoto	Okayama University, Japan	*Hordeum vulgare* and *Brachypodium distachyon*
BIODIVERSITY 4	Th. Zierold	Naturkundemuseum Chemnitz, Germany	*Triops cancriformis* on clay in Finoplast

P.S.S. 1	H. Cottin (PI)	LISA, Paris, France	Chemical samples (comets, Titan, Mars)
P.S.S. 2	C. Szopa	LATMOS, Versailles, France	Chemical samples (Titan)
P.S.S. 3	M. Bertrand	CBM, Orléans, France	Chemical samples (meteorites)
P.S.S. 4	M. Dobrijevic	Observatoire de Bordeaux, France	Biochips
P.S.S. 5	A. Elsaesser	Leiden Observatory, Leiden, Netherlands	Chemical samples (interstellar medium)
P.S.S. 6	G. Baratta	Catania Observatory, Catania, Italy	Chemical samples (comets, graphite)
PDP, BIOCHIP, DEPTH DOSE	T. Berger	DLR, Cologne, Germany	TLDs
R3D-R2	T. Dachev	SRTI-BAS, Sofia, Bulgaria	Active instrument
PPOs	R. Demets	ESA-ESTEC, Noordwijk, Netherlands	PPO passive UV dosimeters

**Table 2:** Amount of samples in EXPOSE-R2.

Experiment	Samples accommodated
BIOMEX	256
BOSS	128
BIODIVERSITY	224
P.S.S.	150
PDP, DEPTH DOSE	46
PPO	24
Total	828

All biological samples were provided by the investigators and accommodated into the sample carriers by MUSC, DLR, Cologne. The chemical samples were provided already mounted in their sample carriers by the PI. The biological experiments BIOMEX, BOSS, and BIODIVERSITY exposed one set of their samples to LEO space conditions in tray 1 and another set to simulated Mars conditions in tray 2. Therefore partly identical samples were accommodated in tray 1 underneath short wavelength transparent MgF_2_ windows and filter combinations and evacuated to space vacuum, and in tray 2 covered with window and filter combinations adjusting the LEO extraterrestrial UV spectrum to a Mars-like spectrum with wavelengths >200 nm and a Mars simulating atmosphere. All passive radiation dosimeters were either stacked into special holes in the 64-well upper sample carriers (DEPTH DOSE) or provided in aluminum holders ready for integration underneath each sample carrier stack on the bottom of the compartments (PDP). The PPO UV dosimeters were stacked into special holes in the filter frames and covered by additional 0.1 MgF_2_ ND filter. With the support of the Payload Developer OHB and ESA, the three trays were fully loaded with all sample carriers and closed on June 12, 2014 in nitrogen atmosphere at the DLR premises in Cologne. The inner nitrogen gas in tray 2 was exchanged with a Mars like gas mixture composed of 95.55% CO_2_, 2.70% N_2_, 1.60% Ar, 0.15% O_2_, ∼370 ppm H_2_O (Praxair Deutschland GmbH) at a pressure of 980 Pa in the PSI at DLR, Cologne on July 2, 2014.

## EXPOSE-R2 MGR Set-Up

For the MGR, a mission identical set of samples was accommodated in three ground trays. As for flight, the trays were closed with the appropriate glassware and inner gas: MgF_2_ glassware and nitrogen gas at ambient pressure for tray 1 and 3 and quartz glassware and Mars gas and pressure for tray 2. The trays were accommodated on a special temperature controlled interface and connected to the vacuum facility PSI 2. For the MGR environmental exposure conditions simulating those experienced by the flight experiment were provided, except for UV < 200 nm, microgravity, and ionizing radiation. For the entire MGR duration pressure and atmospheric composition, temperature fluctuations, and UV 200–400 nm radiation were applied similar to the space flight experiment as far as technically possible. Because the UV sensors on the EXPOSE-R monoblock reused for EXPOSE-R2 never returned to ground for inspection and recalibration, the spectral UV irradiances and fluences were calculated by RedShift for the individual flight samples, using the UV sensor data to verify the underlying models. Final total fluences per compartment were simulated on ground with a SOL 2000 solar simulator (Dr. Hönle GmbH, Germany). The PSI facilities were also extensively used during the pre-flight experiment verification tests and science verification tests to select and verify the most suitable test candidates and experimental set up.

## Mission Sequence

**FIGURE 7:**
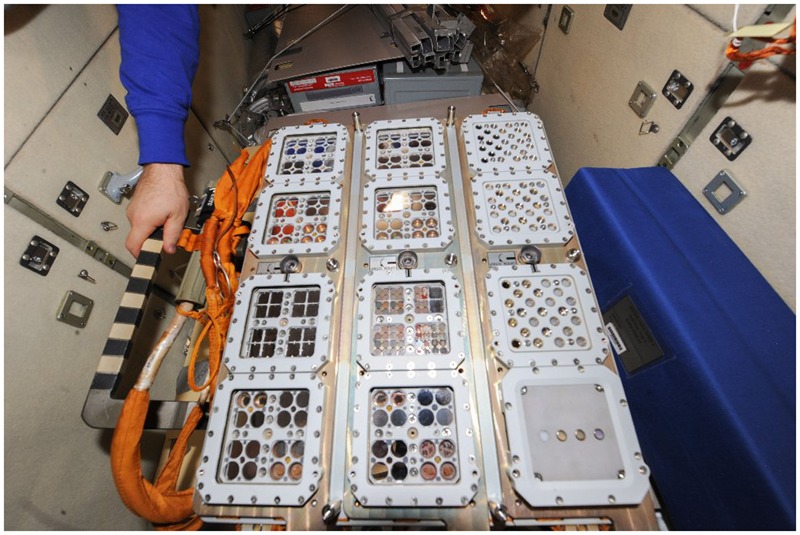
The EXPOSE-R2 trays integrated into the EXPOSE-R monoblock inside the ISS. Credit ESA, ROSCOSMOS.

**FIGURE 8:**
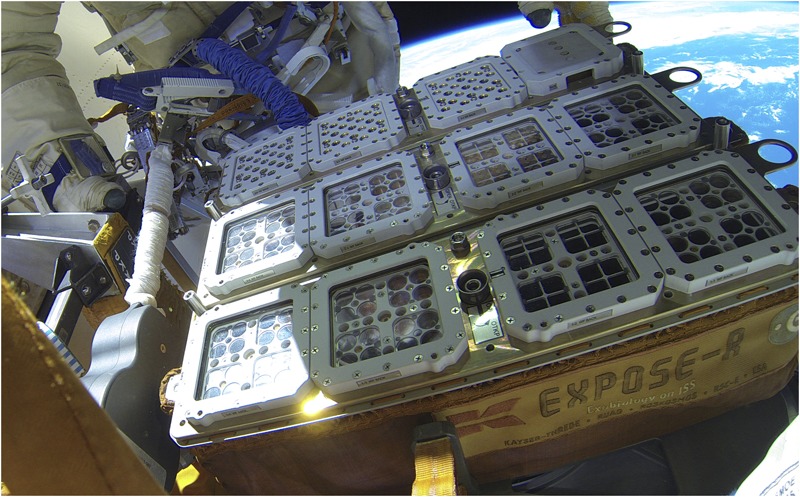
EXPOSE-R2 uncovered in space. The EXPOSE-R monoblock was reused but refurbished with three new trays carrying the samples of the EXPOSE-R2 mission. Credit ESA, ROSCOSMOS.

On July 8, 2014, the three flight trays, sealed and packed in soft pouches and equipped with shock detectors and temperature loggers, were transported in a temperature controlled transport container via Munich and Moscow to Baikonur where they arrived July 23, 2014. Temperature and shock limits were not exceeded during transport. In Baikonur, the trays were accommodated into 56P Progress and launched successfully in the night of July 23, 2014 to the ISS. After docking on July 24, the trays were stored inside the ISS. On August 6, the three trays were inspected by the crew, integrated into the monoblock from EXPOSE-R stored on the ISS (**Figure 7**) and nominal function was confirmed. The now complete EXPOSE-R2 was covered and remained inside ISS until Aleksandr Skvortsov and Oleg Artemyev installed the complete facility on the URM-D platform outside the Zvezda module during EVA-39 on August 18, 2014. On August 20, 2014, valves 1 and 3 of the still covered EXPOSE-R2 were opened by telecommand by Mission Control Center Moscow (MCC-M) marking the start of the vacuum exposure period of the experiment. Data transfer and R3D-R2 functionality were tested. While valve 1 at tray 1 functioned flawlessly, resulting in telemetry data confirming status open, valve 3 of tray 3 telemetry confirmed movement of the valve, but not to the final full open position. Status telemetry nevertheless confirmed valve 3 open and the baroswitches a decrease inside both trays 1 and 3. Therefore, final status “open and evacuated” was confirmed for both trays. Tray 2 remained closed to keep the simulated Mars atmosphere and 980 Pa pressure inside. During the following 62 days EXPOSE-R2 underwent a period of outgassing while protected against solar irradiation by its sun shield. This novel element in the mission operations was introduced after the EXPOSE-R mission, when several optical windows had acquired a discoloration (and thus a loss of transmission) during the spaceflight. The causative factor appeared to be the release of off gassing products from the facility itself and its test samples, which were subsequently photo-processed by solar UV at the inner window surfaces into a thin, semi-opaque contamination layer. To mitigate this unwanted effect on EXPOSE-R’s successor -R2, the outgassing period in space was inserted to remove all volatiles suspected as cause for the window contamination. To avoid overheating of the electronics in this temporary thermally non-optimal configuration, EXPOSE-R2 had to be shut off. No temperature data became available for this period, but Feeder B was programmed to keep EXPOSE-R2 at a temperature above -25°C. After the dark evacuation period for trays 1 and 3 for 62 days, Commander Max Suraev and Alexander Samokutyaev removed the cover from the EXPOSE-R2 trays during EVA-40 (**Figure 8**) on October 22, 2014, starting the UV exposure of the experiments. Power was switched on October 23, 2014 and data were regularly received by telemetry confirming nominal status of EXPOSE-R2.

EXPOSE-R2 was programmed to collect environmental data every 10 s from the six temperature sensors, the four UV sensors, the radiometer, and the R3D-R2 experiment, complemented by health data and status data of the three valves. Data were downlinked regularly to MUSC, DLR via MCC-M. All data were archived, analyzed, and provided to the PIs on an ftp server. They were visualized on the interactive DLR-MUSC webpage (see text footnote 2). During the entire mission, no off-nominal event occurred.

On January 11, 2016, R3D-R2 power off and trays 1 and 3 valves closure commands were sent by MCC-M and acknowledged by EXPOSE-R2. Data received confirmed that the valve of tray 1 was closed, but the valve of tray 3 was not closed. After repeat of the command, EXPOSE-R2 data confirmed execution of the command to close valve 3. Data were thoroughly analyzed by OHB and MCC-M with the agreed conclusion that valve 3 was closed, capturing the space vacuum inside the tray as in tray 1 after 509 days of evacuation to space. After another month, during EVA-42 on February 3, 2016, Yuri Malenchenko and Sergej Volkov returned the complete EXPOSE-R2 facility into the ISS via the PIRS module, ending a total space exposure duration on the URM-D platform of 534 days and full LEO UV radiation for 469 days (ca. 16 months).

Later on ground during tray 3 inspection, it was confirmed that tray 3 valve was not closed properly. Hence, the tray most likely was re-pressurized in an unforeseen, uncontrolled way in the PIRS module on February 3, 2016, ending a 532 days vacuum period for tray 3.

### Tray 3 Return to Ground

Before return to ground, EXPOSE-R2 was stored for 28 days inside the ISS until on March 2, 2016, tray 3 touched down at Karaganda on-board the 44S return capsule at an outside temperature around -3°C. It was packed in a pouch and transport container with temperature and shock monitoring and returned via helicopter to the city of Zhezkazgan in Kazakhstan, from there by plane to Moscow and Munich and finally by car to DLR, Cologne where it arrived in the morning of March 7, at 8:00 h. A first inspection confirmed that shock and temperature limits were not exceeded. Already in Moscow, inspection of the tray 3 baroswitch indicated an inner tray pressure >10,000 Pa, confirming the misgivings of a premature and uncontrolled pressurization of the tray at the end of EVA-42 during return into the ISS. Nevertheless, the valve closed signal retrieved by telemetry was confirmed by tray 3 Electrical Ground Support Equipment (EGSE). After thorough investigation, the reason for the ambivalent information was attributed to a fault in a micro-switch operation.

On March 8, 2016, tray 3 was imported into the anaerobic workbench flooded with nitrogen at ambient pressure at DLR, Cologne. The R3D-R2 instrument was disassembled from compartment 1. When the window frames could be removed from the P.S.S. top carriers and the carriers removed from the tray without previously operating the valve, it was confirmed once more that the tray was already pressurized. The carriers were transferred into a glass desiccator capturing the nitrogen gas. The R3D-R2 instrument was exported from the workbench, inspected, packed, and returned by courier. R3D-R2 arrived safely at the investigators premises on March 16^th^, 2016. The PDP passive dosimeters were also exported from the workbench and directly handed over to the investigator. P.S.S. carriers were removed from the glass desiccator inside the workbench, sealed into plastic bags inside the nitrogen flooded anaerobic workbench at DLR and returned hand-carried together with additional 5°C constant temperature MGR P.S.S. carriers to the PI on March 14, 2016 by CNES.

After de-integration of a part of the P.S.S. sample cells by the PI, the flight P.S.S. carriers with remaining sample cells returned once more to DLR on June 7 for short storage and transportation support to Co-investigators in Berlin. After 22 days storage under nitrogen gas in the anaerobic workbench, the P.S.S. carriers were transported together with the BIOMEX carrier stack of compartment 2 of tray 2 to Berlin on June 29, 2016 upon request of the PIs (see below).

### Trays 1 and 2 Return to Ground

After EVA-42 the remaining two trays were stored in orbit until June 18 for a total of 136 days when they returned to ground with Soyuz 45S. Both trays were stored for return in the cargo compartment underneath the seats of the crew in the Soyuz capsule. Tray 1 was inverted, i.e., with the top window oriented in the same direction as the main g-vector, while tray 2 was oriented in with top windows facing away for g-vector. The latter orientation accelerated the sample toward their carriers. Regardless of the orientation, all samples remained unaffected inside their individual sample wells. The Soyuz capsule landed on June 18, 2016 in Kazakhstan. Temperature at landing site was a sunny 25°C. Similar to tray 3, the trays were packed into bags and transported to Moscow where they arrived on June 19. The first postflight inspection confirmed all valves closed and nominal low inner pressure. The trays were flown to Munich arriving on June 21 and driven by car in the cooling transportation container at 15°C to DLR, Cologne, where they arrived Wednesday, June 22. Shock and temperature limits were not exceeded. After close inspection, both trays were transferred into the anaerobic workbench with nitrogen gas for opening and disassembly of the sample carriers. In order to open the trays, they were equilibrated with ambient pressure nitrogen by opening the valves inside the nitrogen bench via the connected EGSE. All four top window frames and top windows of each tray were removed after unscrewing of the 25 screws attaching each window frame to the tray. Filter frames were unscrewed and removed. Finally, all carrier stacks were unscrewed, removed, and stacked in the original order in a sterile metal box that was used for export from the nitrogen work bench to a sterile biological safety cabinet. The PDP aluminum holders were removed from the bottom of the compartments, also exported from the workbench and directly handed over to the PI. Samples of the BIOMEX 10 experiment from GFZ Potsdam (previously AWI) were oxygen intolerant. Therefore, they were de-integrated from the sample carriers 2-2 directly inside the nitrogen filled anaerobic workbench and transferred into special anaerobic flasks before they were exported from the anaerobic workbench and returned to the investigator. BIOMEX 2 samples from Kyiv, Ukraine, BIOMEX 6 samples from TU Berlin, and BIOMEX 7 samples from DLR, Cologne were also de-integrated inside the workbench form carriers 2-2, exported via the airlock and returned to the respective investigators, leaving the carriers 2-2 with the remaining BIOMEX 8 samples under anaerobic conditions inside the workbench where they were stored in a nitrogen equilibrated glass desiccator until return to the PI together with the stored remaining P.S.S. carriers on June 29, 2016. All other samples of trays 1 and 2 were de-integrated from the sample carriers under ambient but sterile conditions, packed and returned to the individual investigators. For details of Mission and MGR sequnce durations refer to **Tables 3, 4** respectively. Results of the respective experiments are expected to be published in Frontiers in Microbiology under the Research Topic Title: Habitability Beyond Earth and in an upcoming special collection of Astrobiology.

**Table 3:** EXPOSE-R2 mission sequence durations overview.

Mission period	Duration
Inside ISS period launch to EVA-39	26 days
Dark evacuation period valve open to EVA-40	62 days
Valve open to valve closed	509 days
Outside space evacuation period	531 days
Outside data availability	468 days
Total vacuum period tray 3 (closure to PIRS)	532 days^∗^
Total vacuum period tray 1	672 days
Mars gas period tray 2 after second closing	722 days
Outside UV irradiation period EVA-40 to EVA-42	469 days
Outside mission period EVA-39 to EVA-42	534 days
Post-outside storage in ISS tray 3	28 days
Post-outside storage in ISS trays 1 + 2	136 days
Total mission duration tray 3 launch to landing	588 days
Total mission duration trays 1 + 2 launch to landing	696 days

*^∗^With uncontrolled pressurization in PIRS.*

**Table 4:** EXPOSE-R2 MGR sequence durations overview.

MGR period	Duration
Inside ISS period temperature simulation	26 days
Mission temperature simulation according to data	468 days
Total vacuum period MGR tray 3	566 days^∗^
Total vacuum period MGR tray 1	672 days
Mars gas period MGR tray 2	722 days

*^∗^Nominal expected evacuation period.*

## Mission Environmental Parameter

### Temperature Profile during Full Exposure Period

**FIGURE 9:**
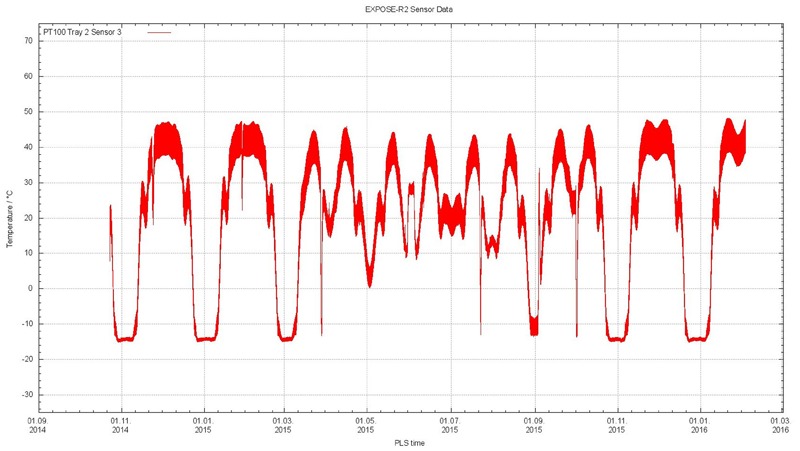
Overview of the EXPOSE-R2 tray temperature profiles for tray 2 temperature sensor 3 and the respective mission period from October 23, 2014 to February 2, 2016 when EXPOSE-R2 was powered and data available. Credit OHB.

Lower limit of the temperature was determined by Feeder B and the associated heating system, preventing a temperature decrease below -20°C. Temperature data from the trays were not available until EXPOSE-R2 was powered after EVA-40 October 23, 2014. For the next 16 months, data were regularly downlinked every month to MUSC via the MCC-M. Highest temperature measured during the mission was 57.98°C at tray 3 compartment 4. Lowest temperature of -20.9°C was detected by the temperature sensor of tray 3 compartment 2. In general, compartments closer to the ISS, i.e., measured by temperature sensors 1-3, 2-3, and 3-3, experienced the higher temperatures during warm periods and were not as cold as compartments facing space during cold periods. A temperature plot for tray 2 temperature sensor 3 for the respective mission period from October 23, 2014 to February 2, 2016, is shown in **Figure 9**: a fast rhythm of temperature fluctuations of approximately 10°C in warm periods and a few degrees in cold periods with a periodicity of approximately 90 min according to day/night times of each orbit were overlaid by approximately 1 month cycles from cold periods reaching -20°C limited by the Feeder B heating system up to 50°C in warm periods. This slow rhythm was due to the changing position of the orbital plane of the ISS w.r.t the sun. From April 2015 to August 2015, these colder phases of the 1 month rhythm reached a minimum of only 0°C with most of the colder periods not decreasing below 20°C. Therefore, a total of 12 warm periods with temperatures of more than 30°C alternated with three cold periods with temperatures below -10°C before the April to August warm period and five periods with temperatures below -10°C after that period.

### UV Irradiation

The total mission average UV-fluence values were calculated by RedShift. To calculate shadow maps for the upper side of EXPOSE-R2, ISS mission flight data of the ISS position in its orbit, ISS attitude, joint angles defining positions of solar arrays and radiators provided by DLR MUSC were used. Evolution of the ISS configuration (3D models), visiting spacecraft and the respective fields of view (FOV) were taken into account for the resulting total mission fluence data for each of the 12 compartments. The UV fluences for the individual compartments and experiments were determined with regard to the different sizes and heights of the samples, leading to different FOVs. As a compromise, the means of the corresponding individually determined FOVs of the samples per compartment were taken. Fluence values for the full spectrum of the extraterrestrial sun as well as for different wavelength ranges were provided. Fluences for the wavelength range from 200 to 400 nm were applied to the respective compartments and experiments during the MGR; the highest fluence was 739 MJm^-2^_200-400__nm_ at compartment 3-2, lowest fluence was 437 MJm^-2^ at compartment 2-1. Note that these fluences reflect the combination of the mean FOV of the samples of the individual compartments and the solar insolation variation over the EXPOSE-R2 surface.

The mean fluence for the biologically active wavelength rage of 200 to 400 nm calculated for EXPOSE-R2 was 536 ± 116 MJm^-2^. The fluences per tray were similar for tray 1 (458 ± 32 MJm^-2^_200-400__nm_) and tray 2 (492 ± 66 MJm^-2^_200-400__nm_), due to the similar geometry of the samples and their FOV. For tray 3, the mean value was 731 ± 12 MJm^-2^_200-400__nm_; sample geometry of tray 3 resulted in an increased FOV and therefore higher fluences compared to trays 1 and 2. Calculated UV data and the underlying model were verified using the UV data of the four UV sensors and the radiometer of the mission period between EVA-40 and EVA-42 when EXPOSE-R2 was powered and not covered. Calculations of the respective UV fluences at sample site underneath each individual optical filter combination for each individual (FOV) and taking into account the UV-attenuating effect of the window “contamination” (see below) together with further detailed information and results on the irradiation of EXPOSE-R2 are expected to be published in the upcoming special collection of Astrobiology by Beuselinck (in preparation).

### Window Inspection

A first visual inspection of the top windows revealed a slight, light-brown discoloration on all MgF_2_ windows of tray 1—referred to as “contamination” in accordance with a similar finding after the EXPOSE-R mission—while the quartz windows of tray 2 seemed clear, except the quartz window on compartment 2-3, also showing a light-brown discoloration. In all cases, the contamination seemed to be less than after the EXPOSE-R mission. This improvement can be attributed to three factors: 1. On EXPOSE-R2, the monoblock of EXPOSE-R was re-used, and had already undergone outgassing in space during its first mission; 2. The period of sun-exposure (with ensuing photoprocessing of volatile materials by solar light) was shorter on -R2 than on -R; 3. EXPOSE-R2 was subjected to an 8-week period of evacuation before solar irradiation was started (see above). Further results on the investigation of this phenomenon are expected to be published in the upcoming special collection of Astrobiology (Demets, in preparation). Data on the solar spectral UV and VIS irradiances from the active R3D-R2 experiment in compartment 3-1 measured every 10 s in addition to ionizing radiation were regularly downloaded together with all other science and status data and provided to the investigator. Publication of the results in the upcoming special collection of Astrobiology is expected by Schuster (in preparation).

### Cosmic Radiation

Cosmic ionizing radiation experienced by EXPOSE-R2 was measure with passive detectors of the PDP and DEPTH DOSE experiments and the active R3D-R2 instrument. Additional thermoluminescence dosimeters (TLDs) were accommodated in some P.S.S. BIOCHIP cells. Preliminary averaged absorbed daily dose rates determined for the three essential components of the radiation field encountered at the orbit of the ISS were: (i) inner Radiation Belt (South Atlantic Anomaly protons) varying from 340 to 844 μGy d^-1^ due to altitudinal changes of the ISS, (ii) galactic cosmic rays (GCR) slowly moving up from 68 to 82 μGy d^-1^ due to the falling solar activity and rising GCR flux over the whole period, and (iii) outer Radiation Belt (electrons) peaking at 2960 μGy d^-1^ (Dachev et al., Results presented during the EXPOSE-R2 Postflight Review, ESTEC, December 2016) (Dachev et al., 2016).

The DOSIS-R2 passive TLDs of the PDP and DEPTH DOSE experiment were distributed over the whole facility in stacks in special cavities between the sample holes of the top UV-exposed sample carriers with very low shielding (down to a few μm/cm^2^) and below the sample carrier stacks at the bottom of the compartments of all three trays. The total mission dose reached values up to 1 Gy (Berger et al., results presented during the EXPOSE-R2 Postflight Review, ESTEC, December 2016). Results are expected to be published in the upcoming special collection of Astrobiology by Berger (in preparation).

### Space Vacuum

The atmospheric pressure outside of the ISS at the Zvezda module was not measured directly by EXPOSE-R2. The Russian agency RSC Energia determined the pressure varying between 1.33 × 10^-3^ and 1.33 × 10^-4^ Pa (10^-5^ and 10^-6^ mm of mercury). The lower pressure usually accounts for the Wake direction (the rear of the ISS relative to flight direction), the higher pressure for the Ram direction (the front of the ISS relative to flight direction).

## The EXPOSE-R2 MGR Performance and Environmental Parameter

All four astrobiology experiments BOSS, BIOMEX, BIODIVERSITY, and P.S.S. participated in the MGR with a flight identical set of samples. The dosimetry experiments were not included in the MGR, because ionizing radiation was not an intended part of the mission simulation. Three ground trays identical to the flight trays with respect to the samples interface were loaded with the flight-identical set of samples and covered with flight-identical optical filtering systems. Inner tray gas and pressure was provided identical as for flight. All three trays were closed under nitrogen atmosphere and the captured nitrogen gas inside tray 2 was exchanged with Mars gas at a pressure of 980 Pa similar to the flight tray 2 with a delay of nearly months with respect to the flight trays end of August 2014. The two year EXPOSE-R2 MGR started with the accommodation of the three trays on the temperature controlled interface on August 28, 2014 and ended on August 16, 2016 with the removal of the trays from the temperature controlled interface of the PSI at DLR. Thereafter, all samples were de-integrated under sterile conditions and similar in sequence as the flight samples and distributed to the investigators for analysis. During the space mission, all data were received in time and without major outages or data loss for the mission simulation.

### Temperature Simulation

Automated temperature simulation for the three MGR trays on the temperature controlled interface started with application of transport temperatures where available with a delay of maximum 2 months due to data analysis and reformatting processes. As soon as environmental data measured and downlinked from EXPOSE-R2 in orbit became available, they were also analyzed and fed into the MGR simulation program of the PSI at DLR, Cologne, controlling the on ground MGR tray temperature simulation according to the data frequency in 10 s steps. After return of the flight trays into the ISS, a Zvezda inner temperature data profile was provided by MCC-M and applied to the MGR samples. Transport data of tray 3 return and trays 1 and 2 return were simulated individually after the appropriate simulated delay times to mimic as best as possible the different flight trays return dates and situations. Flight trays temperatures and MGR temperatures measured at the tray structure (as in flight) deviated by maximum 2°C.

### Vacuum Simulation

On October 15, 2014, MGR trays 1 and 3 were attached to the PSI vacuum facilities and evacuated, simulating the opening of the valves of the respective trays in space and marking the start of the space exposure simulation. Final pressure values of the MGR trays 1 and 3 varied between 1 × 10^-5^ and 6 × 10^-5^ Pa, depending on the simulation temperature. The premature pressurization due to the malfunction of the flight tray 3 valve before return of the trays into the ISS was not simulated. Instead, upon request of the PI, the nominal mission vacuum duration was simulated and the valve of the MGR tray 3 was closed and the tray re-pressurization in nitrogen performed after a total of 672 days.

### UV Irradiation Simulation

As in space, all three MGR trays remained covered light tight for 62 days after evacuation. Thereafter, calculated total mission UV fluences for each compartment provided by RedShift for the wavelengths 200 to 400 nm determined the total MGR simulation irradiation fluences applied using the solar simulator SOL2000 (Dr. Hönle GmbH). The lamps continuous spectrum with wavelengths >200 nm was measured with a calibrated Bentham 150 spectroradiometer (Gigahertz, Türkenfeld, Germany) at the top of the EXPOSE-R2 MGR trays. The MGR compartments were irradiated on ground individually with the same UV fluences for the wavelength range 200–400 nm as determined for the space borne facility. An overview of the EXPOSE-R2 mission and MGR sequence durations with respect to exposure parameters are given in **Tables 3, 4**, respectively.

## Conclusion

EXPOSE-R2 accommodating four astrobiological experiments (BOSS, BIOMEX, BIODIVERSITY, and P.S.S.), the passive dosimeters and the active dosimeter R3D-R2 was launched to the ISS on July 23, 2014. It was installed on the external URM-D platform of the Zvezda module on August 18, 2014. Following a 2-month dark evacuation phase, the experiments were exposed to UV for 469 days until February 3, 2016, when the complete facility was returned into the ISS. The three trays were downloaded with Soyuz in 2 steps due to limited download capacities: Tray 3 returned to ground on March 2, 2016, trays 1 and 2 on June 18^th^, 2016. The EXPOSE monoblock originating from the EXPOSE-R mission again remained stored on board of the ISS for possible further use. During the exposure period, trays 1 and 3 were open to space vacuum and extraterrestrial solar UV radiation >120 nm, while tray 2 kept an inner artificial Mars atmosphere at 1000 Pa pressure and admitted a Mars UV spectrum with wavelengths >200 nm. The mission was carried out in compliance with the requirement of all experiments for (i) space exposure duration of minimum 12 months to maximum 18 months, (ii) storage time after final integration of all samples in the sample carriers until upload of maximum 6 months, (iii) storage on the ISS prior to the space exposure, and (iv) after space exposure of maximum 6 months and v) de-integration until hand over of the samples to the scientists of several weeks. For the complete storage and transportation times, all three experiments requested monitored and controlled temperatures of preferred ambient 20°C with a minimum of 4°C and a maximum of 30°C. During the exposure period, requested upper temperature limits was 60°C. All temperature limits were met. Temperatures experienced on the ISS ranged from -20.9 to 58.0°C, without any individual temperature peak as during the EXPOSE-E mission. In contrast to the two previous EXPOSE missions, no data were lost or corrupted apart from some individual time points.

The selection of appropriate samples, the development of sample design and preparation and the definition of exposure limits were supported by the preflight tests performed in space simulation facilities. In addition, the complete integrated experiment set up for a full mission parallel MGR provided (i) complementing data for comparison and discrimination of effects induced by space parameters, (ii) corrective approaches for the mission simulation experiment as for the P.S.S. experiment in case of off nominal events during the mission, and (iii) a backup in case of major malfunctions or loss of experiments. In general, access to space simulation facilities increases experiment space for increased sample numbers exposed to space conditions, even if they are just simulated, thus providing a valuable additional experiment environment.

Nevertheless, access to space remains irreplaceable for astrobiological investigations and the quest for the limits of life. The complex combination of environmental conditions in space cannot be accurately simulated. Solar simulators can only approximate the short wavelength solar extraterrestrial UV spectrum; simulation of its combination with other space parameters is even less accurate. Therefore, access to space missions for experiments exposing samples to space conditions outside of a spacecraft and with that, outside of Earths protecting atmosphere, ozone layer and—if possible—magnetosphere for prolonged exposure durations remains important.

Though the EXPOSE-R2 mission was an overall success, several issues for improvement for future space missions were identified:

– UV dosimetry. The unavailability of the UV sensors for re-calibration was known in advance. Therefore, UV calculation was procured from the beginning of the EXPOSE-R2 mission, using the UV data only for verifying purposes. Nevertheless, reliable UV fluence measurements are necessary for analysis and interpretation of the data retrieved from the biological and chemical samples.
– Shadowing. Complex carriers like the ISS are by themselves shadow casting obstacles for solar radiation exposure experiments mounted on its external platforms. This leads to a gradient in the insolation over the whole exposure area and to sometimes unexpected shadowing of certain areas (Beuselinck, personal communication) that should be avoided as much as possible.
– Pressure. Pressure data were provided by the Russian operations team, but were not measured at the EXPOSE-R2 facility. For experiments utilizing space vacuum, a pressure detection system at facility site or—preferable—at sample site should be obvious.
– Safety. Safety concerns are a major issue on manned spacecraft. As consequence, selection of test systems is constricted to those that proof to not pose possible hazards to the crew. The accommodation of facilities like EXPOSE on unmanned free flying satellites would reduce the risk of safety problems, provide faster access to space and to a wider range of orbits or even interplanetary routes.

Multiuser exposure facility like EXPOSE are both, a constraint and a benefit for science. While on one side, the experiments accommodated together on one facility by nature have to agree on a common space mission scenario in particular with respect to mission duration, on the other side, the simultaneous exposure allows comparison of results and common use of data as for example those derived from the dosimetry experiments and a higher visibility of the mission.

## Author Contributions

ER, PR, AP, and CP contributed to the scientific support, accommodation, and MGR of EXPOSE-R2. WS, FM, and EJ contributed in all HW and operation design and information aspects. RD was Mission science responsible, PW and RW contributed with Mission operations and respective Information. All authors provided Information in their field to the manuscript and proof read.

## Conflict of Interest Statement

The authors declare that the research was conducted in the absence of any commercial or financial relationships that could be construed as a potential conflict of interest.

## Footnotes

1 http://ooreos.engr.scu.edu/dashboard.htm

2 http://wwwsolar.nrl.navy.mil/solar_spectra.html

3 http://www.musc.dlr.de/expose-r-2/

4 http://www.dlr.de/spacesim

## Abbreviations

EGSE: Electrical Ground Support Equipment
ERA: Exobiology Radiation Assembly
ESA: European Space Agency
EURECA: European Retrievable Carrier
EVA: extravehicular activity, spacewalk
FOV: field of view
Gy: gray
HW: hardware
ISS: International Space Station
LDEF: Long Duration Exposure Facility
LEO: Low Earth Orbit
MgF_2_: magnesium fluoride
MGR: Mission Ground Reference, Mission parallel Ground simulation
MUSC: Microgravity User Support Center
O/OREOS: Organism/Organic Exposure to Orbital Stresses
Pa: Pascal
PI: principal investigator
ppm: parts per million
PSI: Planetary and Space Simulation Facilities at DLR, Cologne, Germany
TLD: thermoluminescence dosimeter
UV: ultraviolet
